# Protoxide of Nitrogen as an Anæsthetic

**Published:** 1869-02

**Authors:** 


### ARTICLE X.

### *Protoxide of Nitrogen as an Anæsthetic.*

A preliminary report of the Committee of the Odontological Society of Great Britian was presented by Mr. W. A. Harrison, Chairman of the Committee, at a crowded meeting of the Odontological Society on Monday evening. By the kind permission of Mr. Harrison, we are enabled to give a digest of the Report; but this we must, however, curtail more than we would otherwise have done, owing to pressure on space.

The Committee, after paying a just tribute to the great service done by Dr. Evans in introducing the gas successfully as an anæsthetic into this country, proceed, in the first place, to consider in detail how far nitrous oxide gas is an efficient anæsthetic. To ascertain this, experiments upon various lower animals were instituted. From these, they arrived at the conclusion that it was free from atmospheric air, a powerful anæsthetic, more rapid in its action, although more evanescent than chloroform and other anæsthetics; and that although, if pushed, it produced death, still the animals were often speedily brought round, when apparently dead, by the admission of air.

They next proceeded to arrive, if possible, at the conclusion whether, as an anæsthetic in man, it was as safe as, or safer than, those in general use. To this they give a guarded answer for the present; stating, however, that it is at least as safe, for short operations, as any other anæsthetic.

They next enumerate the conclusions arrived at, founded on 1380 cases watched and carefully reported on by the various members of the Committee, and on 1051 reported to them on trustworthy authority, as to the advantages and disadvantages of the gas. The advantages are, shortly, these: the rapidity of its effects in producing anæsthesia, the shortest time being twenty-five seconds; rapidity in recovery; its agreeable nature; its being tasteless and less irritating; almost entire freedom from nausea and vomiting, occuring in less than one per cent.; absence of headache and vertigo as a general rule, after complete recovery from the anæsthesia. The disadvantages are noted as consisting in its unsuitableness for long operations, on account of the rapidity of recovery; in the difficulty of making and transporting the gas, and also the expense of the agent; in its being troublesome to make, and requiring unusually complicated apparatus in its administration: in the undesirability of quick recovery in operations followed by much pain; in the administration being occasionally accompanied by twitchings which render it unsuitable for delicate operations.

The Committee next foot up the physiology of its action, with the view to obviate, if possible, any serious results which might follow in its administration. They confess they are as yet unable to explain the *rationale* of its action; but recommend, from experience with lower animals, that, when dangerous symptoms appear, the exhibition be at once suspended, and, should respiration not take place, artificial respiration be resorted to.

The Committee recommend, as the best, most convenient, and cheapest method of procuring the gas in a pure state, the plan of Messrs. John Bell & Co. In its administration, they observe that, whatever instrument is employed, it ought to be as air tight as possible; but they offer nothing fresh, of importance, in this respect, or in the mode of administration. There are, however, a quantity of useful practical details given of considerable interest.

As regards the question, whether there are any special conditions of the system contraindicating its use, they can only say that they have administered, it in persons in various stages of pregnancy in suckling women, in persons subject to asthma, epilepsy, and the like, without any deleterious effects. They, however, advise caution, especially in those affected by disease of the heart, vessels, or lungs. They conclude by drawing the attention of the profession to the success attending the anæsthetic in American by Dr. Colton, and in France by Dr. Evans; and observe that thep propose to prosecute further experiments on the subject, which they hope to lay before the profession at some future time. An appendix of some interesting cases is attached to the Report.

---

